# Supplementary material for: The Impact of Backbone Fluorination and Side-Chain Position in Thiophene-Benzothiadiazole-Based Hole-Transport Materials on the Performance and Stability of Perovskite Solar Cells
Source: Int J Mol Sci. 2022 Nov 2;23(21):13375. doi: 10.3390/ijms232113375 (PMC9654869; doi:10.3390/ijms232113375)
Supplement: Supplementary file 1 [file ijms-23-13375-s001.zip › ijms-1979484-supplementary.pdf]

# Supporting Information

# The Impact of Backbone Fluorination and Side-Chain Position in Thiophene-Benzothiadiazole-Based Hole-Transport Materials on the Performance and Stability of Perovskite Solar Cells

**M. M. Tepliakova [1,*], I. E. Kuznetsov [2], A. N. Mikheeva [1], M. E. Sideltsev [2], A. V. Novikov [1], A. D. Furasova [3], R. R. Kapaev [1,8,9], A. A. Piryazev [2,4,5], A. T. Kapasharov [2], T. A. Pugacheva [2], S. V. Makarov [3,6,7], K. J. Stevenson [4] and A. V. Akkuratov [2]**

[1] Center for Energy Science and Technology (CEST), Skolkovo Institute of Science and Technology,
Nobel St. 3, 143026 Moscow, Russia;
[2] Federal Research Center of Problems of Chemical Physics and Medicinal Chemistry, Russian Academy of Sciences, FRC PCPMC RAS, Academician Semenov Avenue 1, Chernogolovka, Moscow Region 142432, Russia;
[3] School of Physics and Engineering, ITMO University, Kronverksky pr. 49, 197101 St. Petersburg, Russia;
[4] Lomonosov Moscow State University,Department of Chemistry,GSP-1, 1 Leninskiye Gory, 119991 Moscow, Russia;
[5] Sirius University of Science and Technology, Olympic Ave, 1, 354340 Sochi, Russia
[6] Harbin Engineering University, Harbin 150001, China
[7] Qingdao Innovation and Development Center of Harbin Engineering University, Qingdao 266000, China
[8] Department of Chemistry, Bar Ilan University, Ramat Gan 5290002, Israel
[9] Bar-Ilan Institute of Nanotechnology and Advanced Materials, Ramat Gan 5290002, Israel

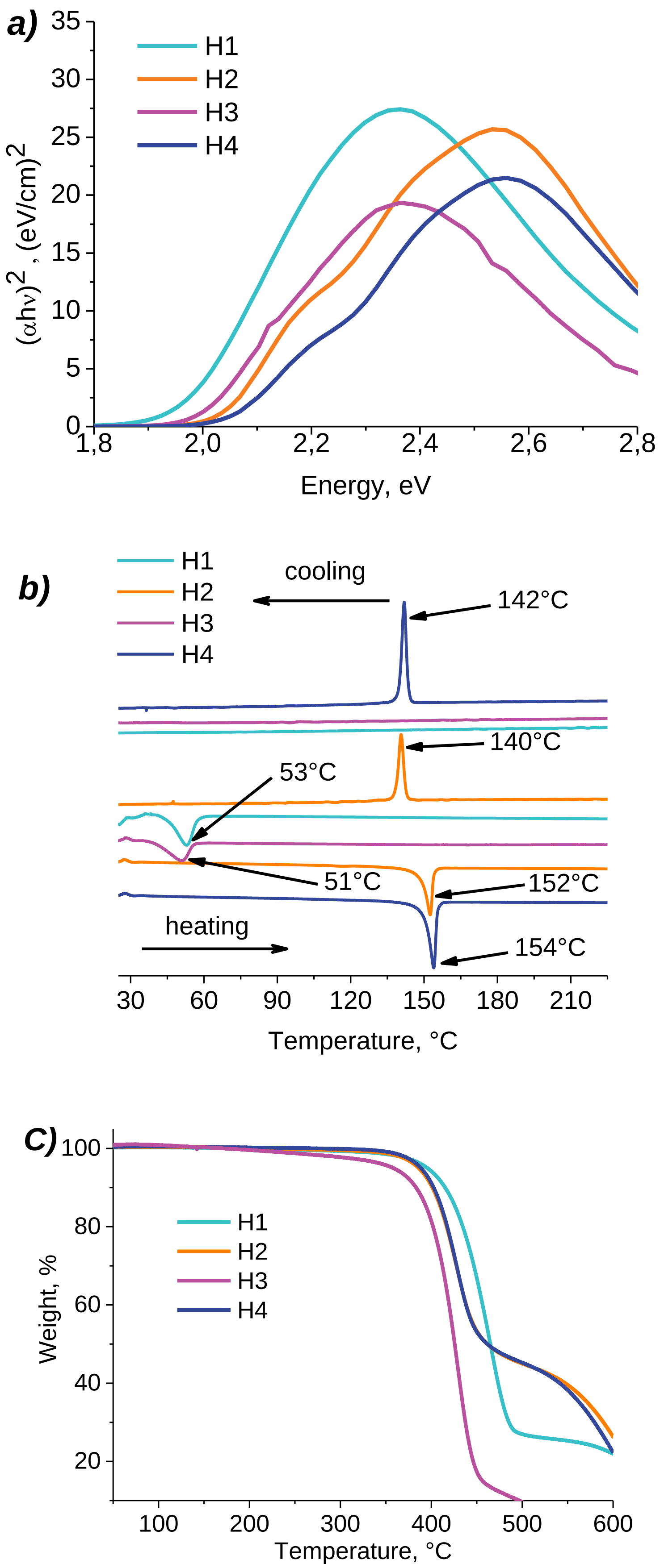


**Figure S1**. Tauc plots (*a*), DSC curves (*b*) and TGA *(c)* for **H1-4**

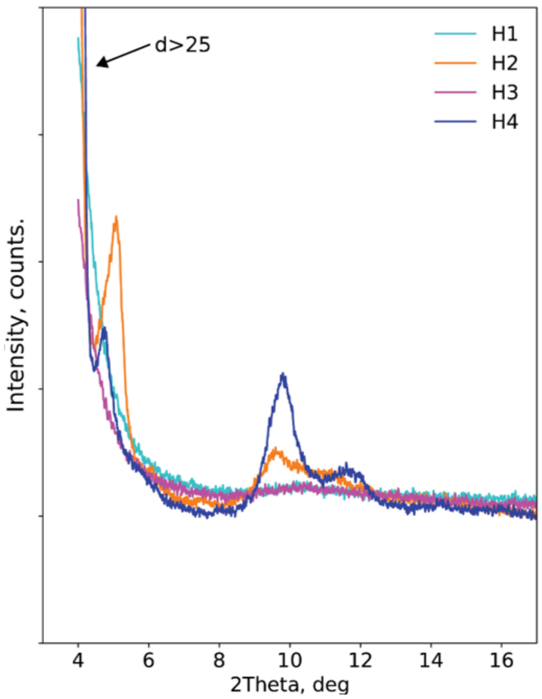


**Figure S2.** Selected regions of the XRD patterns of **H1-4**

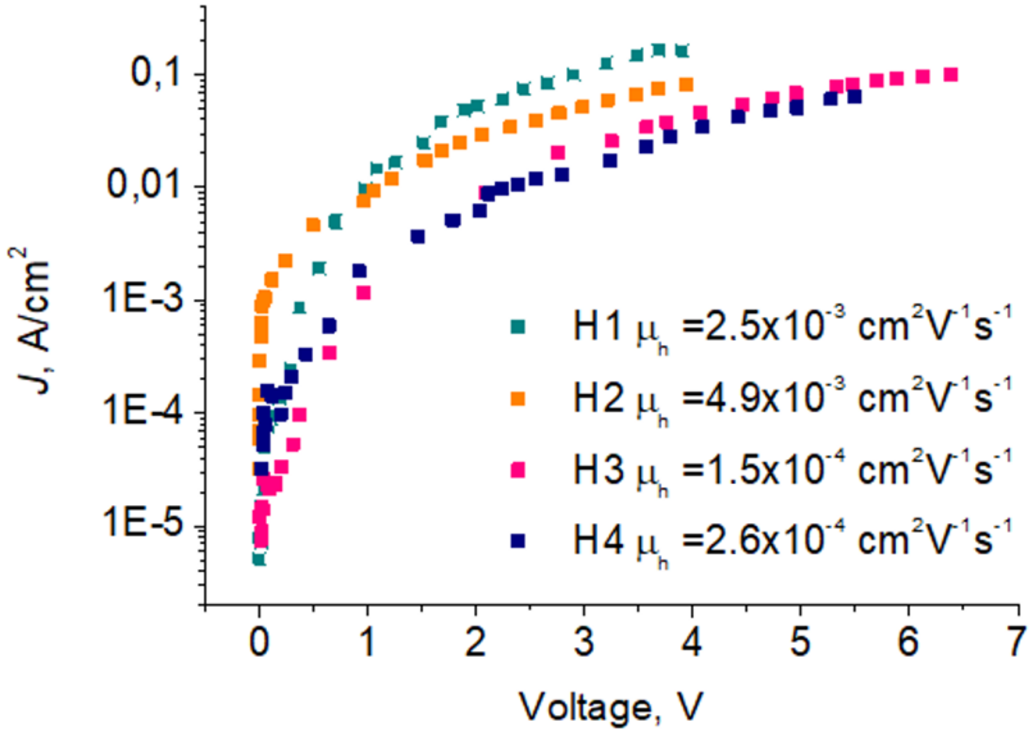


**Figure S3.** J-V curves of hole-only devices based on compounds **H1-H4**

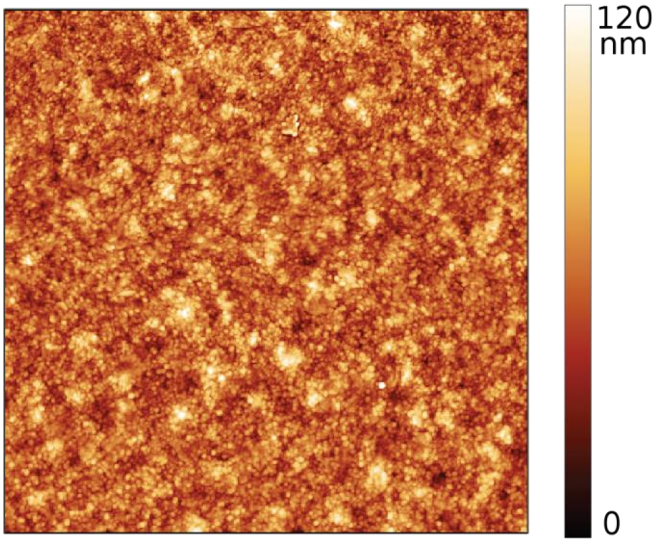


**Figure S4.** 30×30 μm AFM scan of bare perovskite layer.

| HTM | $V_{OC}$, mV | $J_{SC}$, mA cm$^{-2}$ | FF,% | PCE,% |
|---|---|---|---|---|
| Spiro-OMeTAD | 910±60 | 16.1±0.5 | 46±10 | 7.0±2.0 |

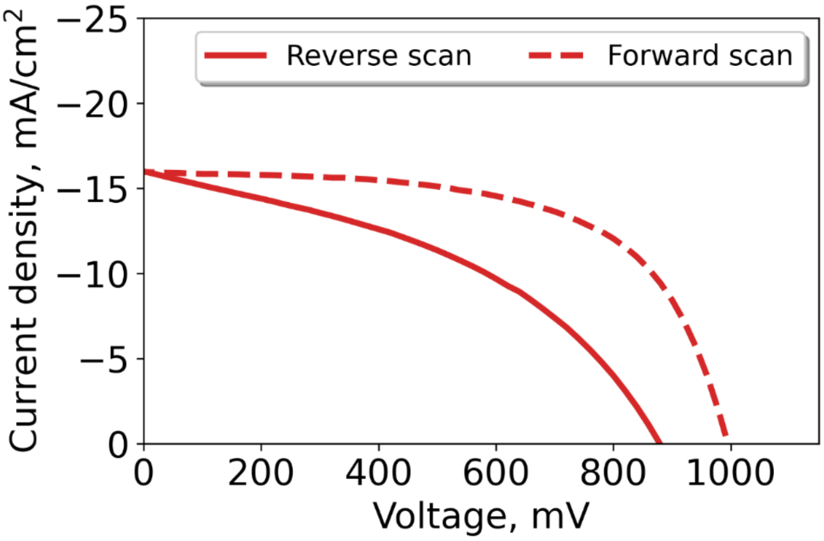


**Figure S5**. *J*-V curve for PSCs with spiro-OMeTAD as HTM.

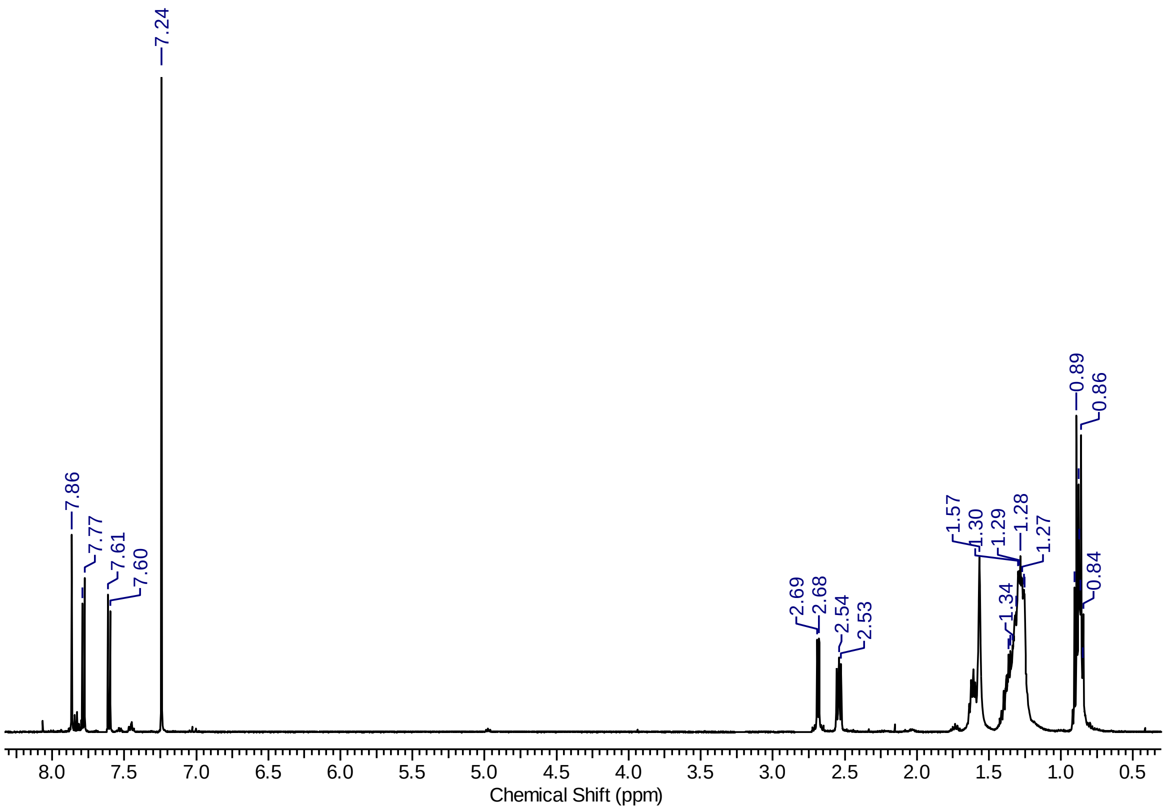


**Figure S6**. $^{1}$H NMR spectrum of compound **2a**

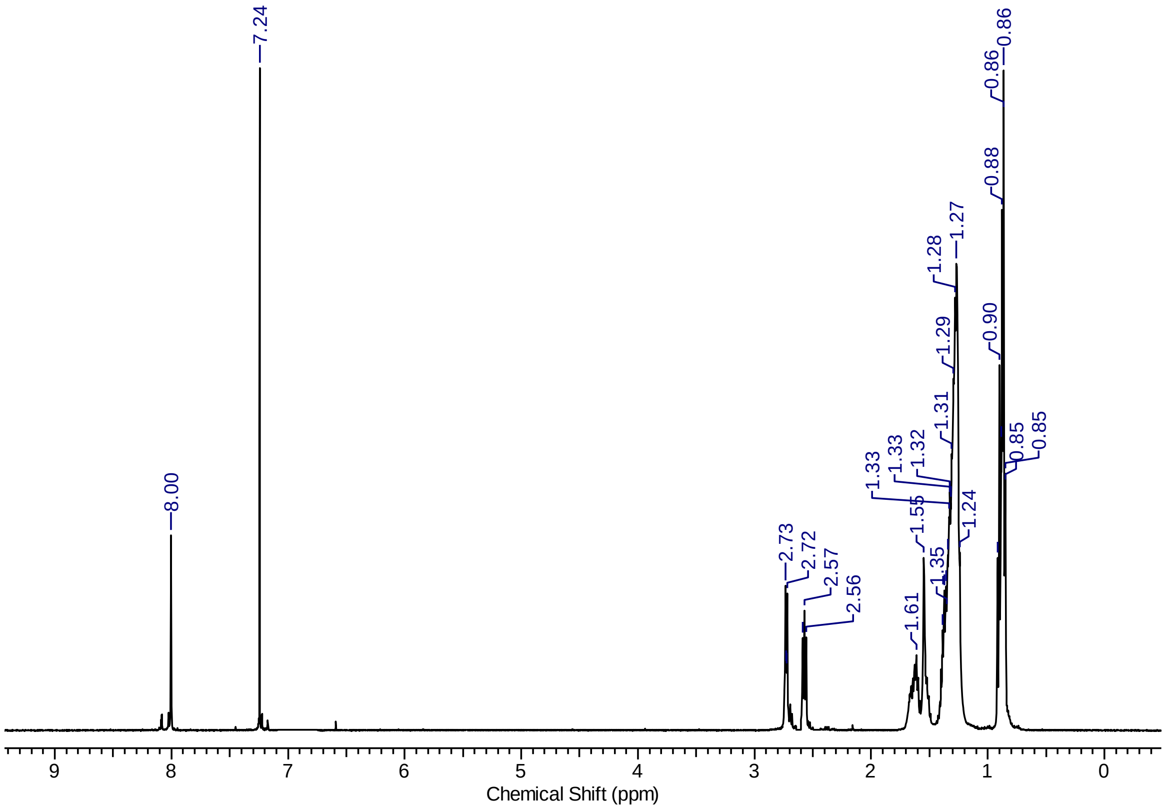


**Figure S7**. $^{1}$H NMR spectrum of compound **2b**

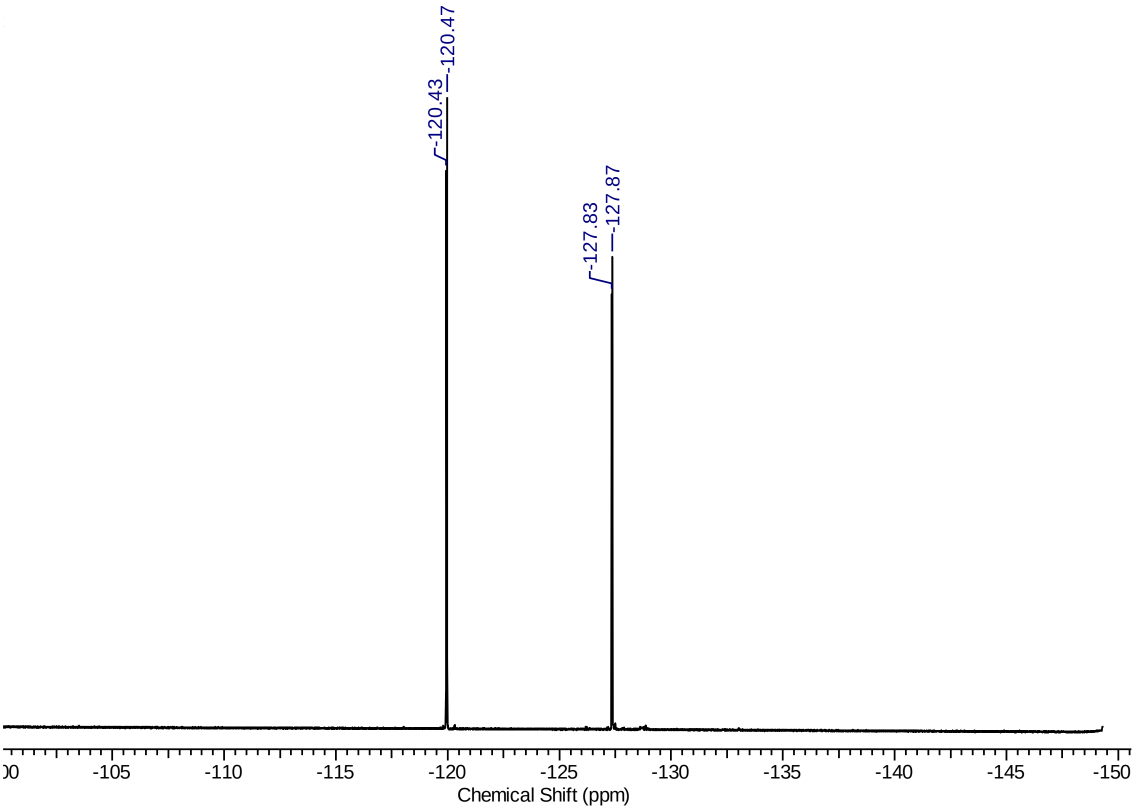


**Figure S8**. $^{19}$F NMR spectrum of compound **2b**

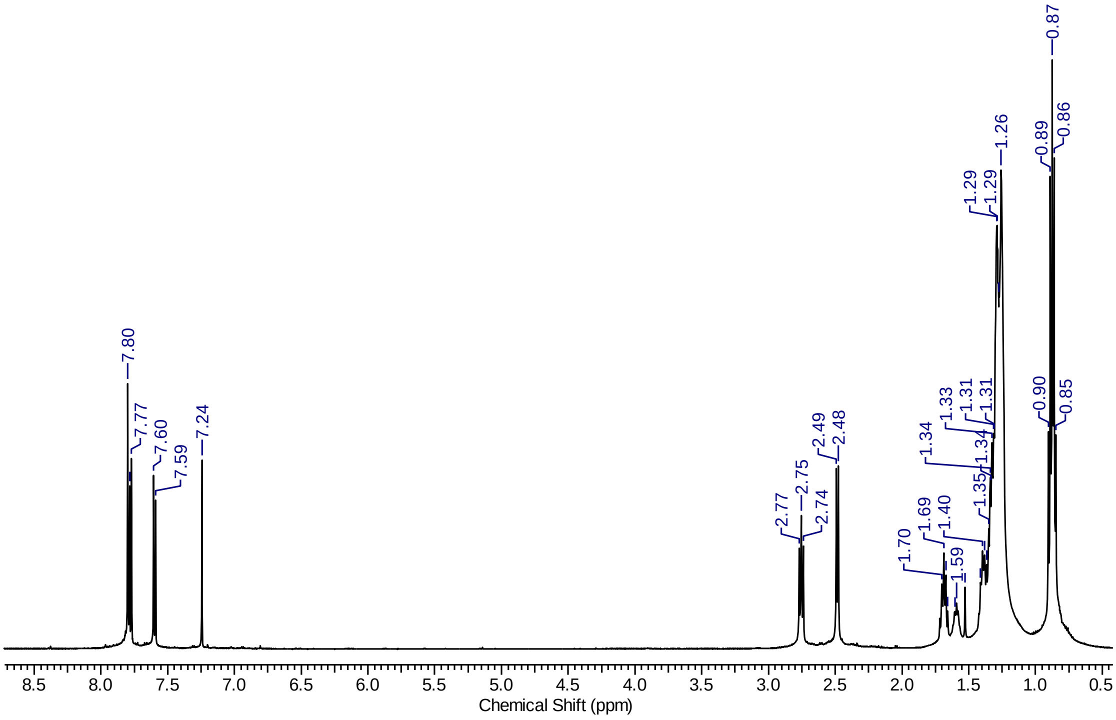


**Figure S9**. $^{1}$H NMR spectrum of compound **2c**

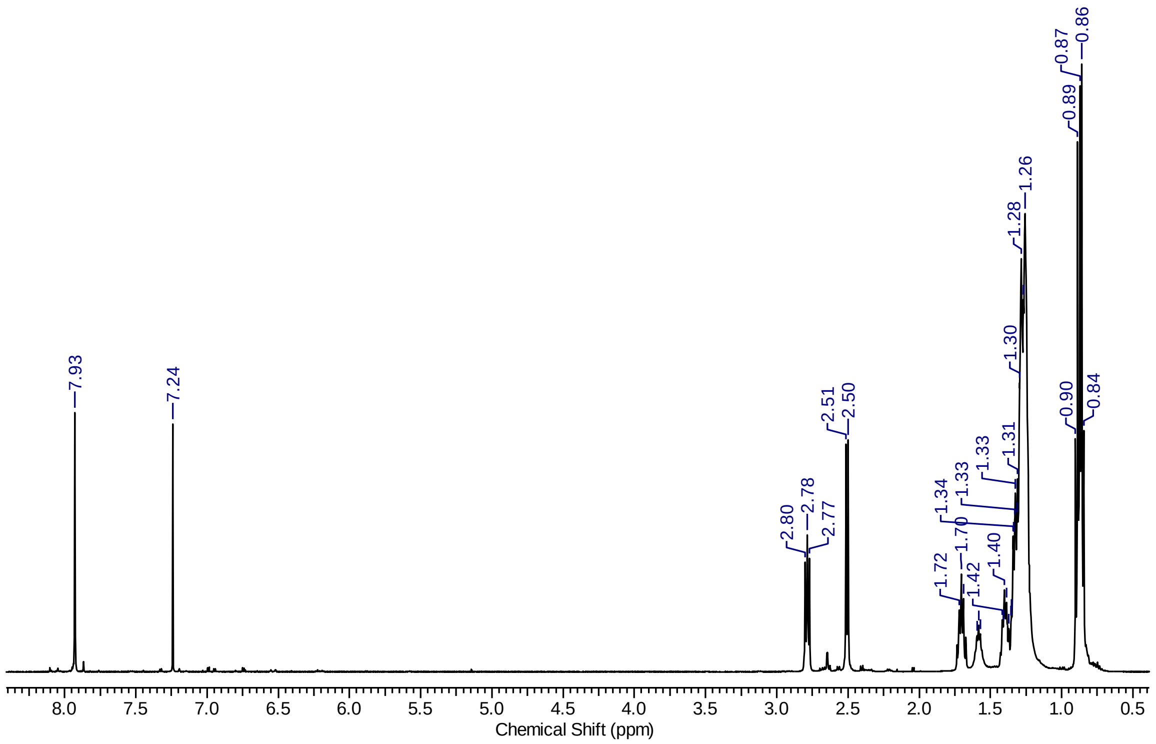


**Figure S10**. $^{1}H$ NMR spectrum of compound **2d**

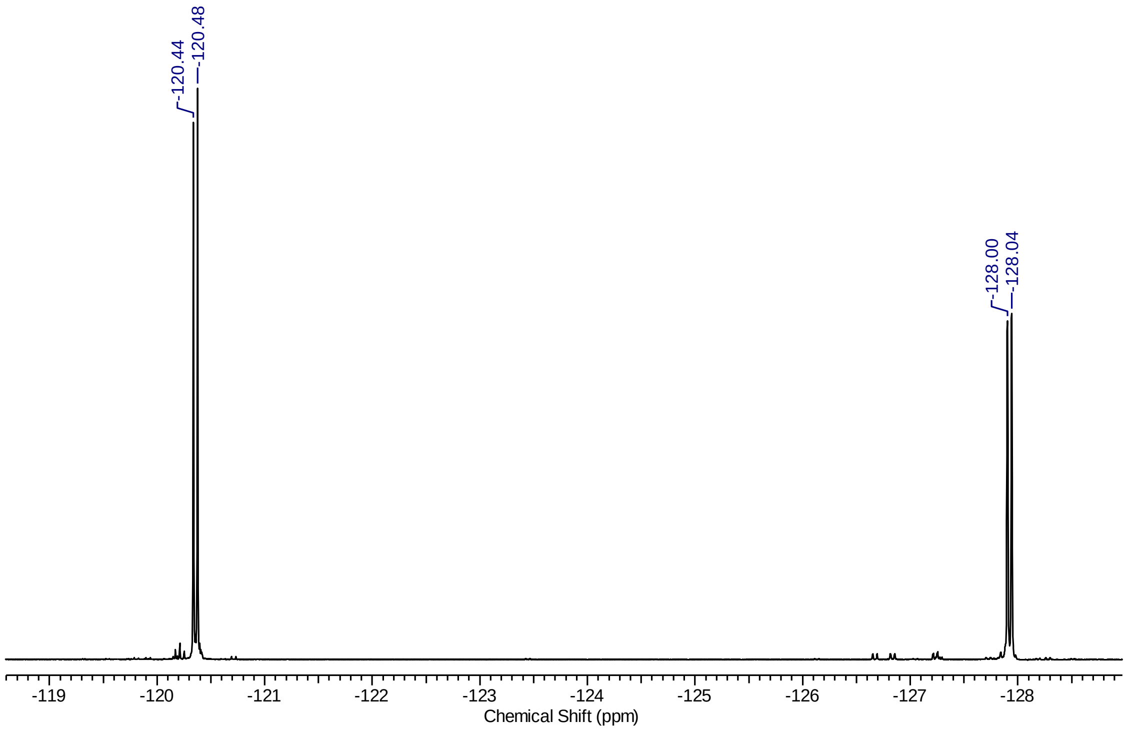


**Figure S11**. $^{19}F$ NMR spectrum of compound **2d**

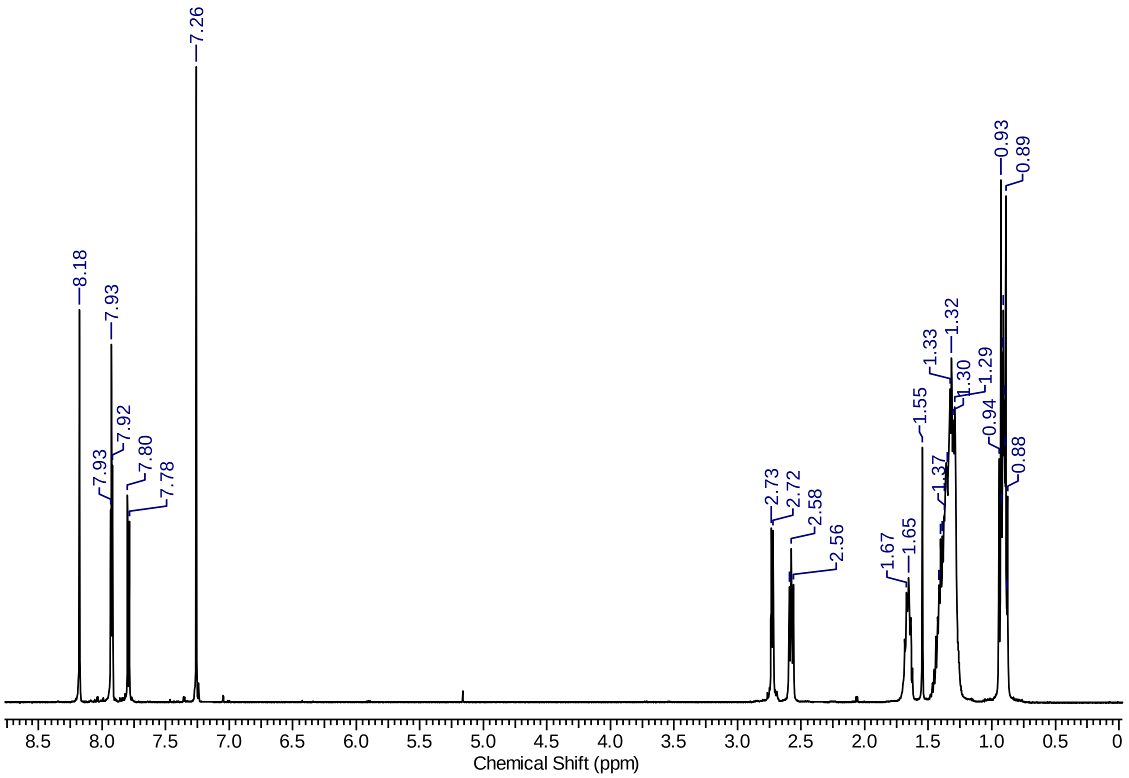

**Figure S12**. $^{1}$H NMR spectrum of compound **H1**

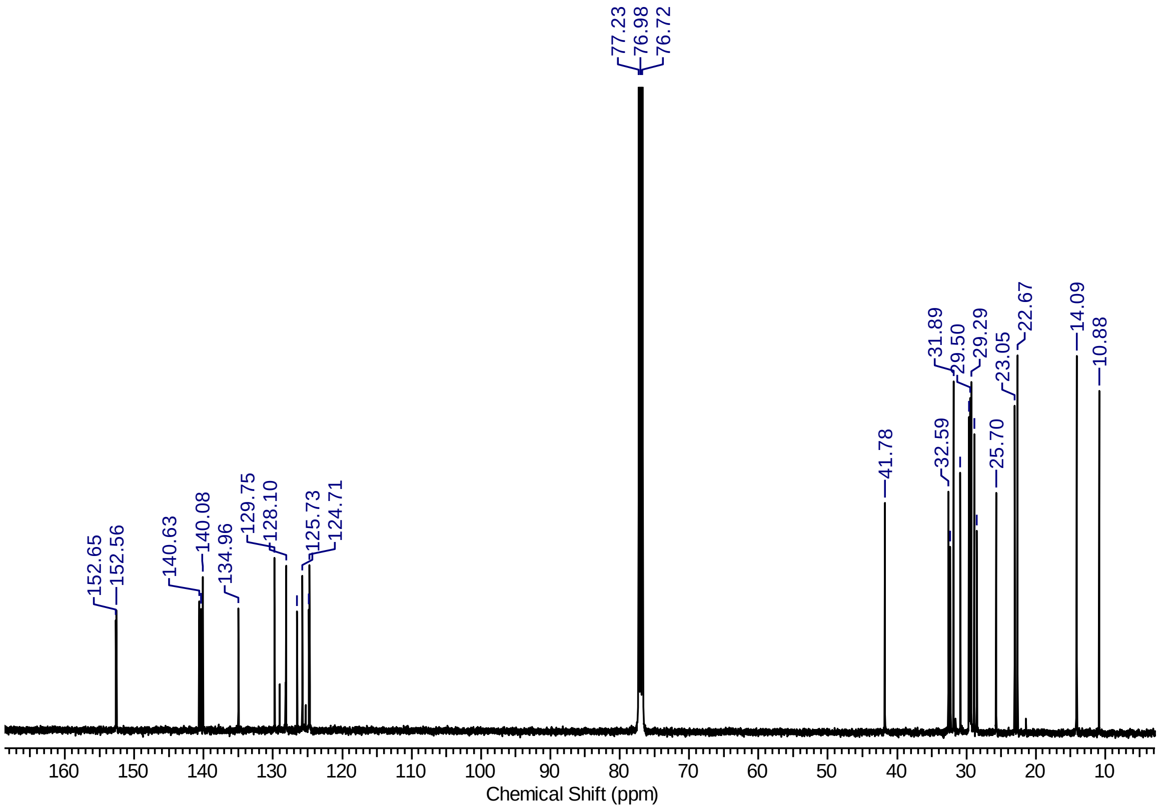

**Figure S13**. $^{13}$C NMR spectrum of compound **H1**

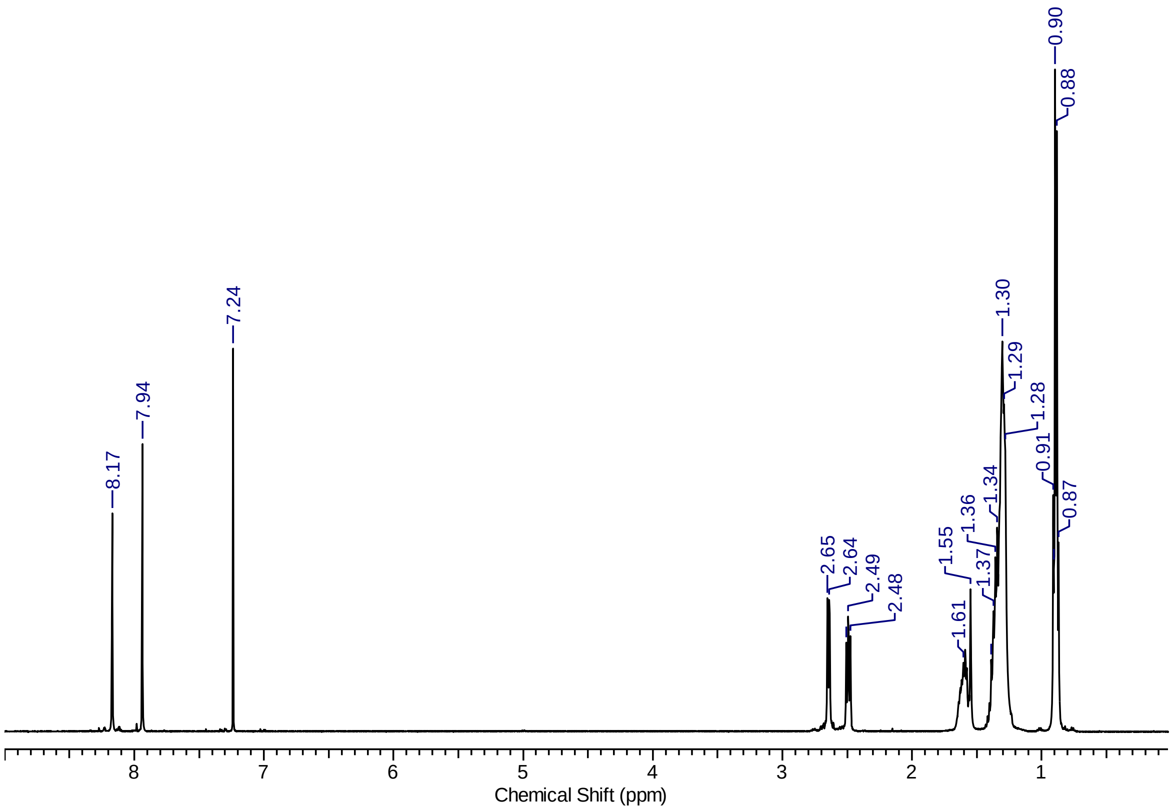


**Figure S14.** $^{1}H$ NMR spectrum of compound **H2**

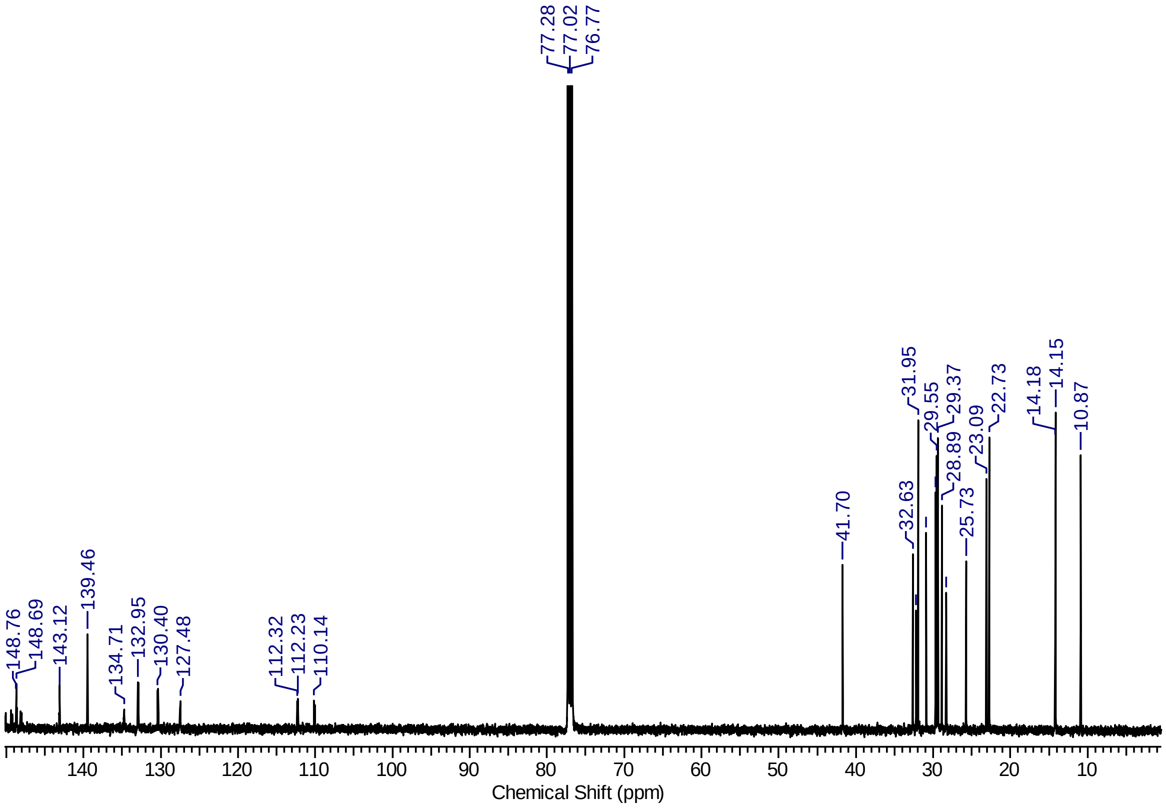


**Figure S15.** $^{13}C$ NMR spectrum of compound **H2**

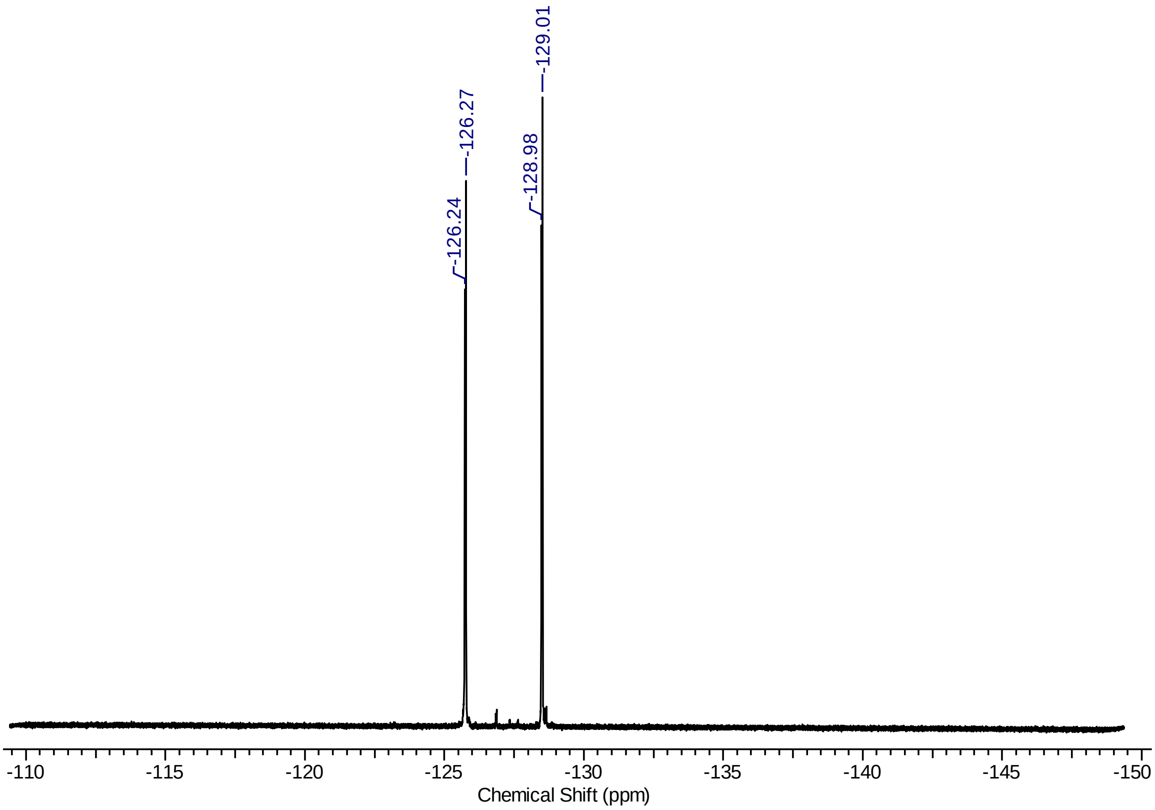


**Figure S16**. $^{19}$F NMR spectrum of compound **H2**

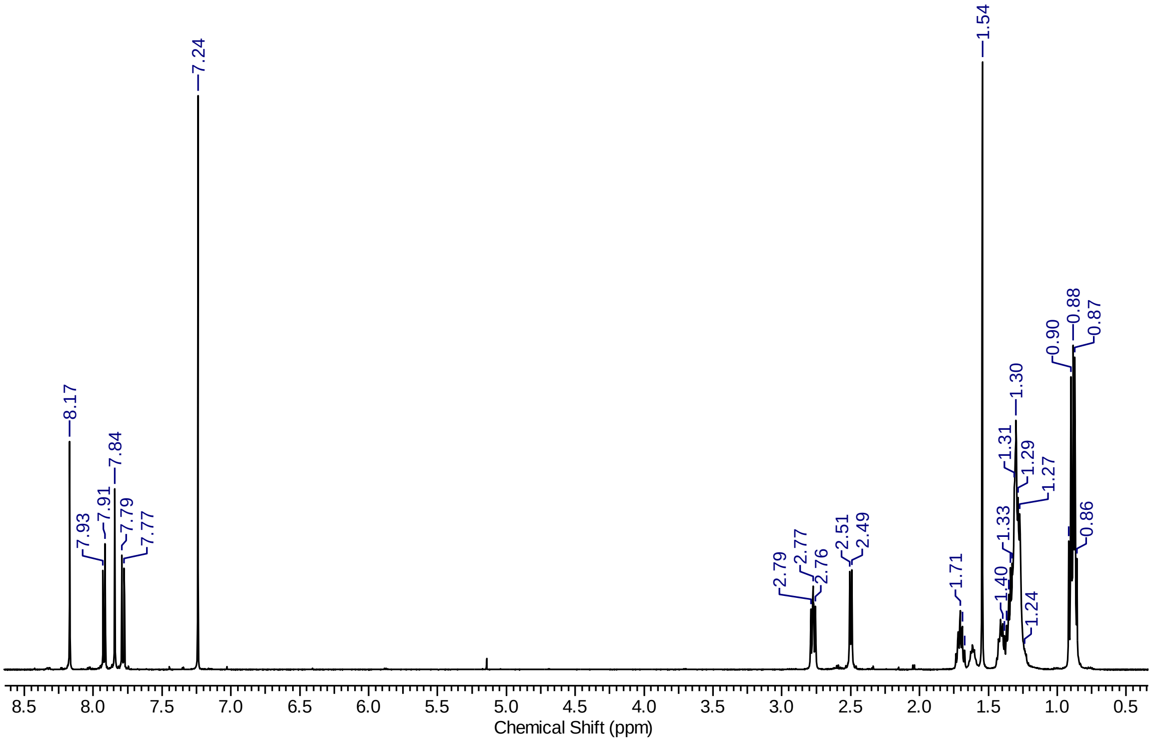


**Figure S17**. $^{1}$H NMR spectrum of compound **H3**

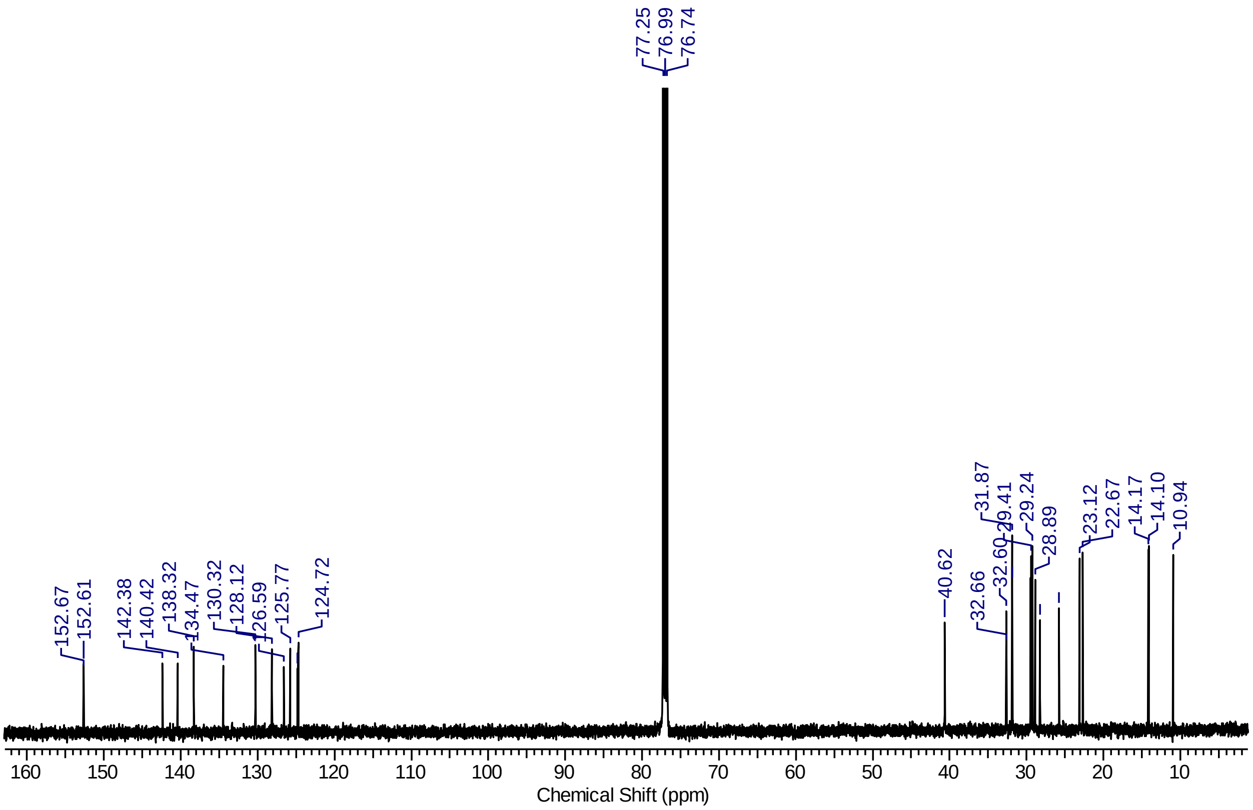


**Figure S18**. $^{13}C$ NMR spectrum of compound **H3**

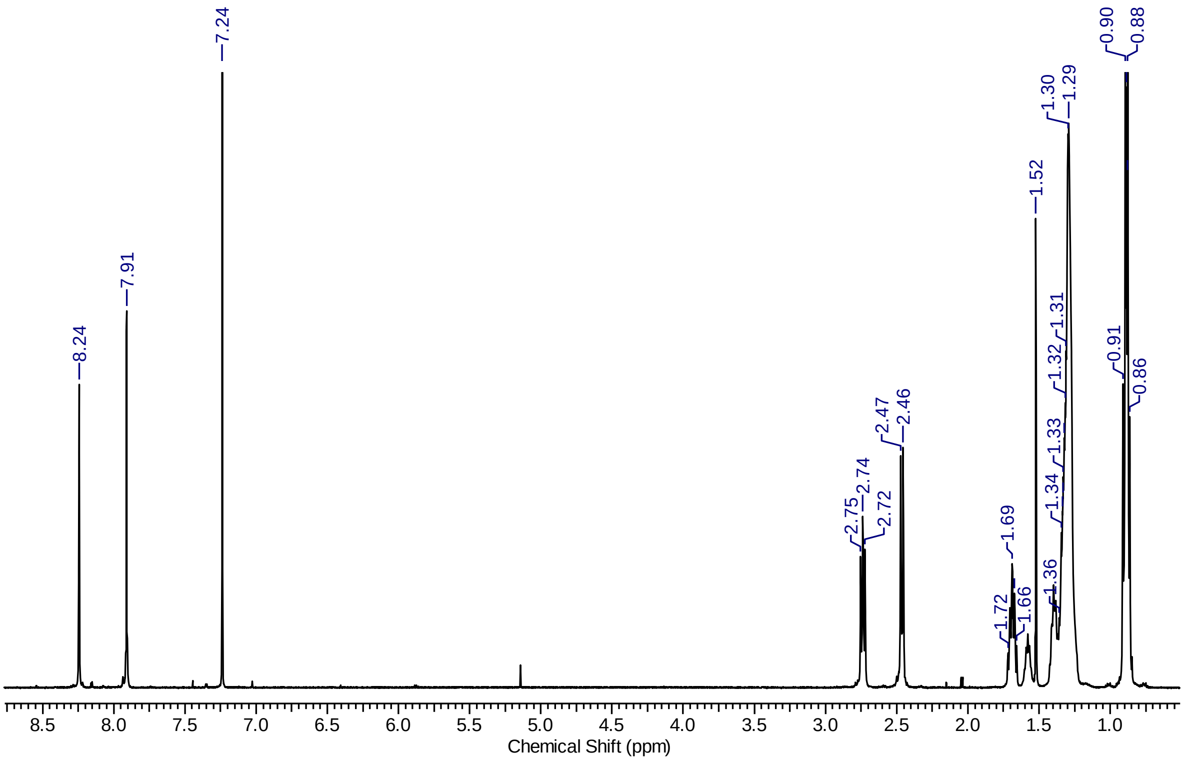


**Figure S19**. $^{1}H$ NMR spectrum of compound **H4**

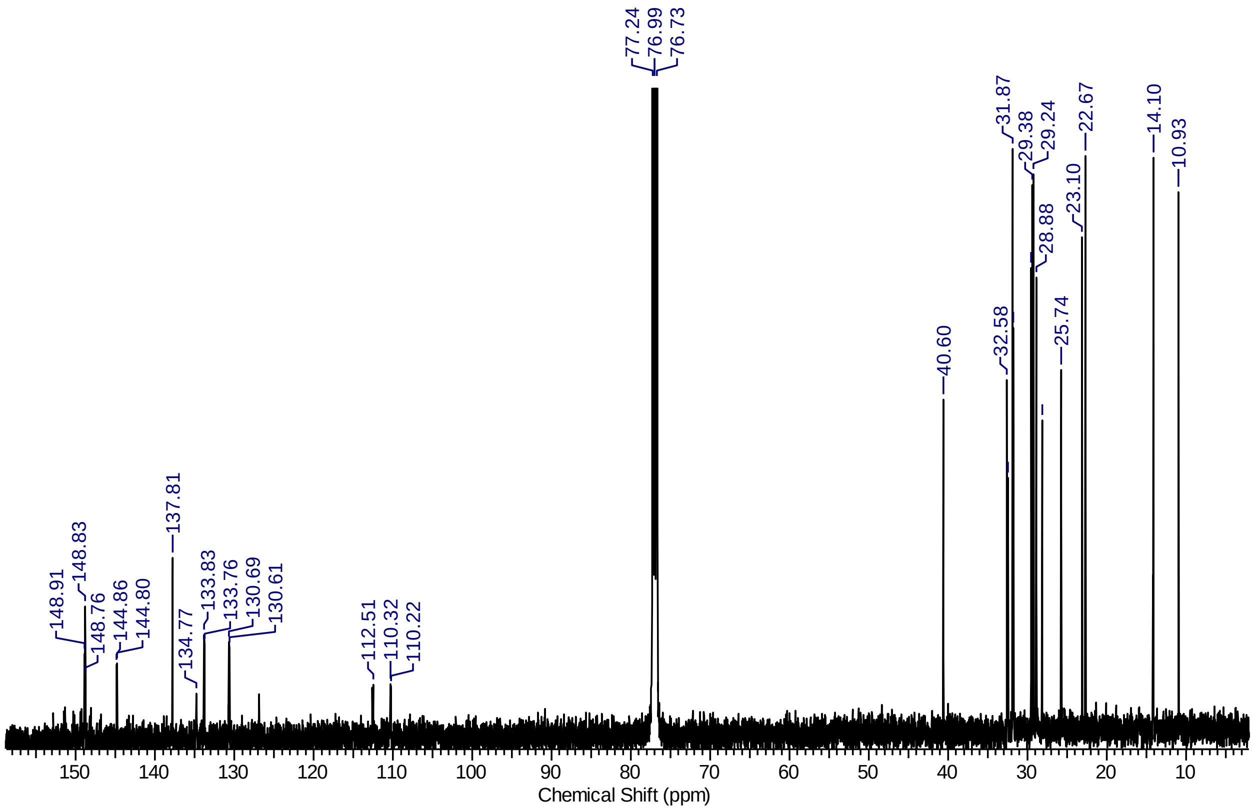


**Figure S20**. $^{13}C$ NMR spectrum of compound **H4**

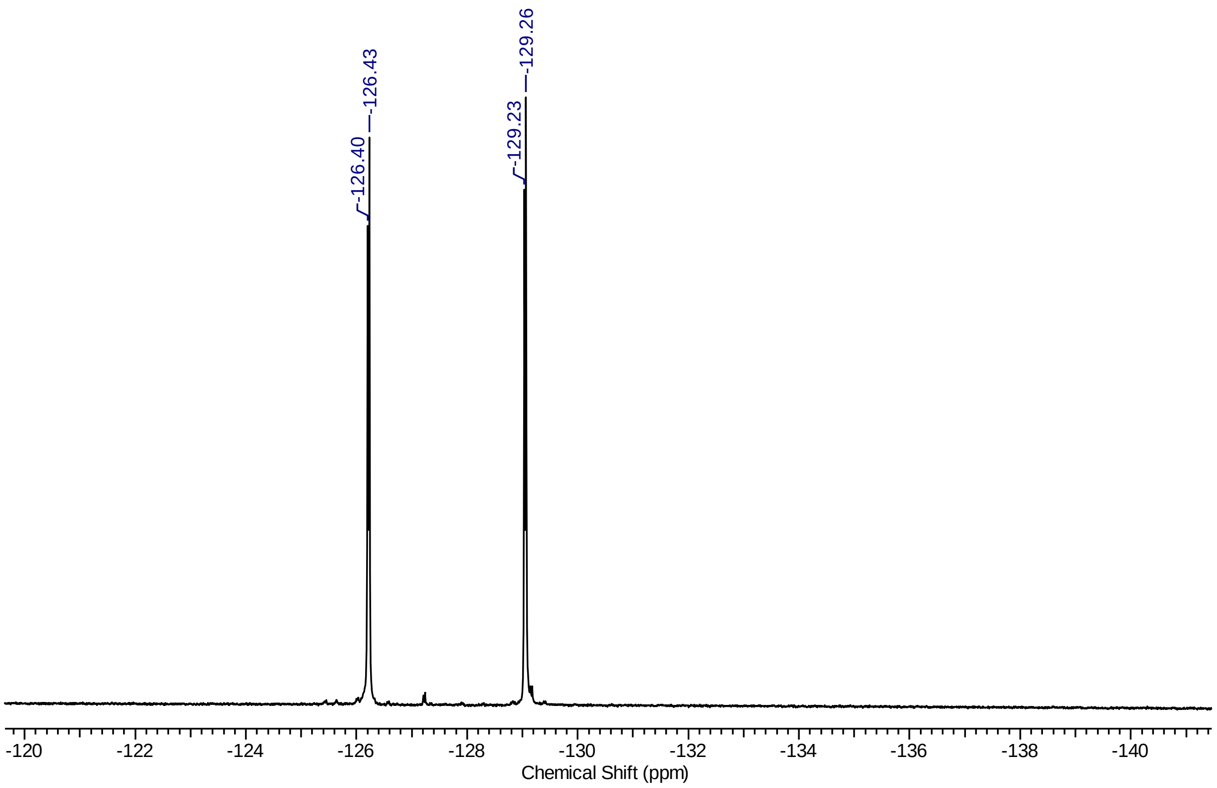


**Figure S21**. $^{19}F$ NMR spectrum of compound **H4**
